# Re-Framing the Knowledge to Action Challenge Through NIHR Knowledge Mobilisation Research Fellows Comment on "CIHR Health System Impact Fellows: Reflections on ‘Driving Change’ Within the Health System"

**DOI:** 10.15171/ijhpm.2020.02

**Published:** 2020-01-15

**Authors:** Jo Rycroft-Malone, Joe Langley

**Affiliations:** ^1^Faculty of Health and Medicine, Lancaster University, Lancaster, UK.; ^2^Lab4Living, Art & Design Research Centre, Sheffield Hallam University, Sheffield, UK.

**Keywords:** Embedded Researcher, Knowledge Mobilisation, Evidence, Fellowship, Co-Production

## Abstract

The ambition of the Canadian Institutes for Health Research Health System Impact (HSI) Fellowship initiative to modernise the health system is impressive. Embedded researchers who work between academia and non-academic settings offer an opportunity to reframe the problem of evidence uptake as a product of a gap between those who produce knowledge and those who use it. As such, there has been an increasing interest in the potential of people in embedded research roles to work with stakeholders in the co-production of knowledge to address service challenges. In this commentary, we draw on research and experiential evidence of an embedded researcher initiative, which has similar intentions to the HSI Fellowships programme: the National Institute for Health Research (NIHR) Knowledge Mobilisation Research Fellowship (KMRF) scheme. We outline the similarities and differences between the two schemes, and then consider the work, characteristics and skills, and organisational arrangements evident in operationalising these types of roles.

Despite ever increasing attention, resource and research effort, how to best support a knowledge-based health and care service delivery system, particularly at scale, remains frustratingly elusive. As such, the ambition of the pan-Canadian Health System Impact (HSI) Fellowship initiative that aims to “drive change and modernize the health system”^1^ is impressive. This initiative provides a deliberate attempt to build capacity and capability within the health system through the development of individuals in roles that co-locate in service and academic institutions – as ‘embedded researchers.’ This idea of embedding researchers in these boundary spanning or intermediary roles is in part a response to how the challenge has traditionally been framed, ie, that the problem of evidence use and uptake is a consequence of a gap between those that produce knowledge and those that use it. Embedded researchers who work within non-academic contexts but have an affiliation with academia, in theory, offer an opportunity by reframing this problem. Rather than filling a gap between producers and users, the focus is on considering all stakeholders as producers and users of different forms of knowledge, and to carry out work to co-produce (co-create and use) knowledge.

As noted by Sim et al,^1^ internationally there is increasing attention on intermediary or embedded researcher type roles^2-5^ in efforts to bring knowledge to bear on particular service challenges. An increased focus on the potential of embedded researcher roles is also reflective of a turn to co-productive ways of working, and the infrastructure, capacity and capability required to support this way of working.^6^

As eluded to by Sim et al,^1^ and other commentators,^7,8^ the establishment of embedded roles, the development of the individuals who take them on, and the infrastructure required to support them requires considerable thought and investment. Additionally, enacting these roles is not without challenge. As Vindrola-Padros et al^2^ note, dual affiliation and role strain, building trusting relationships whilst maintaining critical distance, and being constrained by host organisation’s when there are negative or harmful research results are issues that need constant attention and negotiation. Thus far, despite the increasing popularity of establishing these roles, we argue that there has been perhaps too little attention on their evaluation, with some exceptions.^9-11^ Here we reflect on an English embedded researcher type initiative that has had a longer history than HSI (2012-2017), but had similar ambitions called the National Institute of Health Research (NIHR) Knowledge Mobilisation Research Fellowship (KMRF). In reflecting on the KMRF scheme, we draw on the research evidence base about embedded researchers and experiential evidence of a KMRF (JL).

## Knowledge Mobilisation Research Fellowships

Box 1 describes the five key objectives of the KMRF scheme.

The first round of the scheme was called Knowledge Mobilisation Fellowships, and focused on mobilising research evidence into practice (Objectives 1, 3-5; Box 1). From round two, research [‘R’] was introduced (Objective 2, Box 1). This addition was to ensure that the fellowships were about doing *and* researchingknowledge mobilisation. Fellowships were funded in a competitive process based on the quality of their plans for the development of self, doing knowledge mobilisation, researching knowledge mobilisation, developing capacity in National Health Service (NHS) organisation to mobilise research evidence and building service involvement in research.

**Box 1. KMRF Scheme Objectives**

1. To build capacity by developing individuals who can lead and champion knowledge mobilisation for NIHR funded research and other applied health research
2. To improve and share the research-informed evidence base around knowledge mobilisation activities through new research
3. To improve the uptake, application and influence of NIHR funded research and other applied health research within the NHS
4. To develop capacity in NHS organisations that contributes to knowledge mobilisation research evidence
5. To improve the quality and relevance of NIHR research through greater service involvement

Abbreviations: KMRF, Knowledge Mobilisation Research Fellowship; NIHR, National Institute of Health Research; NHS, National Health Service.

A maximum of five fellowships were awarded in each annual competition. Table 1 summarises annual numbers of applicants, fellowships awarded, and total investment.

**Table 1:** KMRF Awarded

	**2012**	**2013**	**2014**	**2015**	**2016**	**2017**	**Total**
Number of applicants	39	15	10	10	23	19	116
Number of KMRF awards	5	5	3	5	5	3	26
Total investment (£)	748 709	1 043 053	728 059	1 143 524	1 054 362	698 852	5 416 558

Abbreviation: KMRF, Knowledge Mobilisation Research Fellowship.

There are both similarities and differences between HSI and KMRF schemes. The main similarities are that both have an objective to *do* some kind of translational work, ie, putting evidence into practice. Additionally, both HSI and KMRF expect dual host organisational set up, with one being an academic organisation and the other being a service delivery organisation. However, there are some differences, which are summarised in Table 2.

**Table 2:** Differences Between HSI and KMRF Schemes

**KMRF**	**HSI Fellowship**
Smaller initiative with a maximum of five awards a year made, and 26 in total between 2012 to 2017.	HSI Fellowship awarded 95 fellowships in total between 2017 to 2018.
KMRFs are not restricted to early career researchers. Professors have been awarded KMRFs.	HSI Fellowships are restricted to doctoral and post-doctoral scholars.
KMRFs have explicit objective to do research about Knowledge Mobilisation.	HSI Fellowships do not have an explicit emphasis on undertaking their own research.
KMRFs do not have a structured training programme. Fellows defined their own as part of the funding process. Individual KMRFs came together to create their own peer group but there was no imperative, organisational support or prompting from the funder.	HSI Fellowships have structured training programme in predetermined ‘core competencies’ and have a peer network with organisational structure and purpose.
KMRF have specific objective to build capacity in knowledge mobilisation in the healthcare provider organisations.	HSI Fellowships have objective to work with healthcare provider organisations to implement or use research but not specifically to develop capacity or capability in the organisation.
KMRF objective is to develop individuals who can be knowledge mobilisation champions but does not relate this to a career path.	HSI Fellowships objective is to develop individuals for HSPR fields through experiential learning within contexts of practise – there is a reference to ‘impact orientated’ career paths.
KMRFs had varied backgrounds, not exclusively in health services research.	HSI Fellowships have strong background in health research including doctoral foundation.

Abbreviations: KMRF, Knowledge Mobilisation Research Fellowship; HSI, Health Services Impact; HSPR, Health Services and Policy Research.

Whilst the ambition of the initiatives is broadly similar, as Table 2 shows, the parameters of the two schemes were different in that the KMRF competition appears to have been more open in terms of, for example, applicants’ backgrounds. As such, the cohort of 26 KMRFs represent a variety of backgrounds and therefore approaches to the embedded role from the more traditional health services research^12^ to the more co-creative.^13^ Despite a difference in approach, the unifying feature was an expectation that the KMRFs work with or alongside those in health and care services.

## The Work

Sim et al^1^ touch on the work done in their embedded researcher roles, including the co-production of outputs to support practice innovation and improvement. Given the aim of the HSI fellowship is to bridge the knowledge practice gap, it is assumed that the ability to work across boundaries through developing and maintaining partnerships with different people and communities would be particularly critical to success. Bridging or spanning boundaries episodically (for example for specific tasks), as well as blurring them through a more continuous approach to being embedded in day-to-day activities^9^ and being critical friends^14^ requires considerable effort and work. As Cassidy et al^7^ suggest, this will require the HSI fellows to ‘attune to the relationship components embedded within the programme.’ The need to attend to relationships is also evident in the ongoing research into embedded researchers of Ward and colleagues (https://www.embeddedresearch.org/uploads/8/0/2/1/80213224/ukkmbf.3.pdf) which identifies one of the features of embedded research initiatives as ‘Relational Role.’ In their conceptualisation, Relational Role includes the independence of the embedded researcher and their approach to providing input.

‘Authentic collaboration, partnership and engagement provides a context for action’^6^ for those occupying embedded roles, however this requires careful navigation to align expectations and to work across boundaries. It also requires an ability to work with different types of evidence. For example, with a background of design engineering JL uses design practises as a means of discovery and research. As part of his knowledge mobilisation fellowship he has explored whether design practises, which take the form of eliciting, articulating and synthesising knowledge, and embodying it in material objects (prototypes) are useful for co-creating knowledge. His research suggests the practice of ‘Collective Making’ has additional value in the way it can manage relationships and power dynamics.^13^ Personal dynamics such as dominant personalities, being introvert or extroverts, and social and structural dynamics such gender, age, discipline and education levels can be negotiated through ‘making’ because:

1. As an activity it is equally ‘out of the norm’ for all parties, when used in research contexts; it therefore a leveller.
2. ‘Making’ and ‘playing’ are associated with childhood, hobbies, and activities people choose to do for pleasure and leisure, as such they take away hierarchies and give permission to be less formal.
3. The process changes language and modes of thinking being less reliant on words, which enables a different and collective way of communicating.

Brokering relationships and increasing the flow and use of knowledge through social contact^14^ also requires those in embedded roles to, as JL describes it, ‘fill in the gaps’ and provide ‘leadership between the cracks.’ Filling in the gaps refers to the work required to manage different tasks, activities and people, which are dynamic and shifting as the work progresses leaving spaces that need connecting for maintaining forward momentum. Whilst leadership between cracks refers to a type of informal leadership required to work with and between disparate individuals and groups to drive motivation and action.

## Characteristics and Skills

Given the work of those in embedded roles, people working in them need to embody a variety of characteristics and skills. In a review of published and grey literature of the knowledge, skills, and attitudes of people^15^ working in knowledge translation roles, a number of core competencies were identified, which are summarised in Table 3.

**Table 3:** Core Competencies

**Knowledge**	**Skills**	**Attitudes**
Understanding the context	Collaboration and teamwork	Confidence
Understanding the research process	Leadership	Having trust
Knowing how knowledge is disseminated	Sharing knowledge	Valuing research
Being aware of evidence resources	Knowledge synthesis	Self-directed lifelong commitment to learning
Understanding knowledge translation and evidence-based practice processes	Dissemination of research findings	Valuing teamwork
	Use of research findings	
	Fostering innovation	
	Knowledge brokering	
**From Grey Literature**		
Quality improvement methods and tools	KT planning	Integrity
Communication strategies	Project management	Commitment to professional work ethic and professional behaviour in interaction with internal and external contacts
Health policy and systems	Information technology use	Commitment to high standards of professionalism
	Sound judgment	Interest in the developments in communications
	Discretion/tact/diplomacy	
	Resourcefulness	

Experientially, and perhaps as a function of a design engineering background, JL identified less with the knowledge components, and more with skills and attitudes. This highlights that embodying these embedded roles is partially a function of the person’s background, but also about how that aligns with what is needed for the role at the time, in that context, and with those people. In fact, over time it has become apparent to JL that knowledge synthesis and brokering were inherent to his way of working because it is the modus operandi of a designer. Presenting himself as a design engineer and as naïve to the clinical contexts of those he was working with was an advantage because it removed assumptions about a baseline of knowledge for all participants. The idea of credibility being a function of the characteristics of the person, rather than their level of knowledge is also evident from other research.^14,16^ For example, it might in some circumstances be advantageous for an embedded researcher to be a non-clinician, because being at a distance from the clinical challenge enables a focus on the research process. However, this runs counter to Sim et al’s^1^ reflections in which they identify their current or former health professional background knowledge and experience as helpful for supporting networking and relationship building for effective collaboration.

We argue that approaches to identifying potential embedded researchers be guided, but not constrained, by a list of specific competencies or particular background. As noted by others^6^ being able to work productively with different constituencies in dynamic contexts requires transferable qualities such as being comfortable with messiness, a good communicator with different audiences, being flexible, being able to manage conflict and being tenacious and creative.

## Organisational Arrangements

Sim et al^1^ describe the role of a HSI fellow as a ‘central agent’ who navigates the health system to become a ‘conduit for system level change.’ As part of these arrangements, the fellows work alongside decision makers in the health system whilst maintaining a link to academia. It is less clear in their reflection and their Framework for understanding the HSI Fellow as an Embedded Researchers how and what particular organisational arrangements and contexts facilitate role enactment. Within England, the Collaborations for Leadership in Applied Health Research and Care (CLAHRC) have provided an organisational arrangement for the development and support of a number of different types of embedded researcher type roles. In later rounds of the KMRF scheme, the CLAHRCs where host organisations for individual fellows. CLAHRCs were a distributed regional service and academic partnerships funded to increase applied health research and the use of research in practice. Evidence from evaluations of CLAHRCs^14,16-19^ have demonstrated the pivotal role that embedded researcher type roles played in developing the partnerships themselves, as well as in co-producing research and knowledge mobilisation through inhabiting the worlds of service and academia.

However, there were features of CLAHRCs that were more facilitative of individuals operating in these embedded roles.^16,18^ For example, the existence of matched funding incentivised health service and academic organisations to work together, and helped to ensure role holders had dedicated resources, including protected time. Second, CLAHRCs that positioned their strategy and approach towards evidence co-production in contrast to a knowledge transfer approach created contexts more conducive to enacting an embedded role because they were a better cultural fit. Finally, CLAHRCs that were structured and organised in a way that facilitated connection between people and organisations, as opposed to silos, enabled easier navigation across boundaries.

Beyond the generalisable benefits of the host organisation attributes described above, there will be idiosyncratic benefits for each individual embedded researcher, some of which will be emergent and serendipitous whilst others can be carefully considered in advance to get the best ‘fit’ between embedded researcher, proposed work and host organisation. The work of Ward and colleagues (https://www.embeddedresearch.org/uploads/8/0/2/1/80213224/ukkmbf.3.pdf) is prompting people to do this, particularly from the perspective of the researcher.

From experience, JL’s ‘home’ was in Yorkshire & Humber CLAHRC’s Translating Knowledge to Action theme, which had a strong focus on co-design and coproduction, and was therefore a good fit for someone with a design background. This CLAHRC was a conducive context for operating as a knowledge mobilisation fellow because there were already brokered and trusted relationships with service provider organisations and a rich organisational know-how of working together. This context was a ‘ready-made’ collaborative and trusted network for JL to navigate, and a context in which to facilitate the introduction of new ways of working such as ‘collective making.’^13^ Within this context JL benefitted from relative autonomy from service and academic affiliations and agendas. This context was also more conducive to sustaining an individual in an embedded researcher role as the funding and host arrangements ensured an appropriate support structure.

## Conclusion

The potential contribution of embedded research type roles in the co-production of knowledge towards service improvement and transformation is evident, and as such the numbers, and associated labels for such roles, is increasing. Specific initiatives such as the Canadian HSI Fellowships and England’s KMR Fellowships also demonstrate national funders’ commitment to the potential of this way of working. We have highlighted some similarities and differences between these schemes, and drawn on research and experiential evidence to show that people operating in these embedded researcher roles have to navigate complex environments and tailor their action accordingly. Their success is likely to be mediated by how flexibly and appropriately they can draw on a varied toolbox and personal skill set, and by the organisational arrangements, that is, resources and culture, which support them. However, whilst there is an increase in embedded roles there has not been a corresponding growth in systematic and large-scale research about the mechanisms and impacts of these roles. This is now a gap that needs to be filled.

## Acknowledgements

JL received funding from the NIHR Knowledge Mobilisation Research Fellowship scheme between 2014 and 2017.

JRM was a member of the NIHR Knowledge Mobilisation Research Fellowship funding panel from 2012 to 2017.

## Ethical issues

Not applicable.

## Competing interests

Authors declare that they have no competing interests.

## Authors’ contributions

JRM and JL co-created the manuscript. Both read and agreed the final version.

## Authors’ affiliations

^1^Faculty of Health and Medicine, Lancaster University, Lancaster, UK. ^2^Lab4Living, Art & Design Research Centre, Sheffield Hallam University, Sheffield, UK.
